# Occurrence of *Serratia marcescens* Carrying *bla*_IMP-26_ and *mcr-9* in Southern China: New Insights in the Evolution of Megaplasmid IMP-26

**DOI:** 10.3390/antibiotics11070869

**Published:** 2022-06-28

**Authors:** Yuxia Zhong, Wanting Liu, Peibo Yuan, Ling Yang, Zhenbo Xu, Dingqiang Chen

**Affiliations:** 1 Microbiome Medicine Center, Department of Laboratory Medicine, Zhujiang Hospital, Southern Medical University, Guangzhou 510282, China; yuxia0301@163.com (Y.Z.); lwtjyk2022@163.com (W.L.); pbyuanpb@163.com (P.Y.); 2 Department of Laboratory Medicine, The First Affiliated Hospital of Guangzhou Medical University, Guangzhou 510120, China; jykresearch@126.com; 3 School of Food Science and Engineering, Guangdong Province Key Laboratory for Green Processing of Natural Products and Product Safety, Engineering Research Center of Starch and Vegetable Protein Processing Ministry of Education, South China University of Technology, Guangzhou 510640, China; 4 Department of Civil and Environmental Engineering, University of Maryland, College Park, MD 20742, USA; 5 Research Institute for Food Nutrition and Human Health, Guangzhou 510640, China

**Keywords:** *Serratia marcescens*, multidrug resistance, *mcr-9*, *bla*_IMP-26_, megaplasmids IMP-26, evolutionary pathway

## Abstract

The spread of multidrug-resistant enterobacteria strains has posed a significant concern in public health, especially when the strain harbors metallo-beta-lactamase (MBL)-encoding and mobilized colistin resistance (*mcr*) genes as such genetic components potentially mediate multidrug resistance. Here we report an IncHI2/2A plasmid carrying *bla*_IMP-26_ and *mcr-9* in multidrug-resistant *Serratia marcescens* human isolates YL4. Antimicrobial susceptibility testing was performed by the broth microdilution method. According to the results, *S. marcescens* YL4 was resistant to several antimicrobials, including β-lactams, fluorquinolones, sulfanilamide, glycylcycline, and aminoglycosides, except for amikacin. To investigate the plasmid further, we conducted whole-genome sequencing and sequence analysis. As shown, *S. marcescens* YL4 possessed a circular chromosome with 5,171,477 bp length and two plasmids, pYL4.1 (321,744 bp) and pYL4.2 (46,771 bp). Importantly, sharing high similarity with plasmids pZHZJ1 and pIMP-26, pYL4.1 has an IncHI2/2A backbone holding a variable region containing *bla*_IMP-26_, *mcr-9*, and two copies of *bla*_TEM-1B_. After comprehensively comparing relevant plasmids, we proposed an evolutionary pathway originating from ancestor pZHZJ1. Then, via an acquisition of the *mcr-9* element and a few recombination events, this plasmid eventually evolved into pYL4.1 and pIMP-26 through two different pathways. In addition, the phage-like plasmid pYL4.2 also carried a *bla*_TEM-1B_ gene. Remarkably, this study first identified a multidrug-resistant *S. marcescens* strain co-harboring *bla*_IMP-26_ and *mcr-9* on a megaplasmid pYL4.1 and also included a proposed evolutionary pathway of epidemic megaplasmids carrying *bla*_IMP-26_.

## 1. Introduction

*Serratia marcescens* is a bacterium of the *Enterobacteriaceae* family that thrives in damp environments such as water and soil and can survive for months on inanimate surfaces. The bacteria were thought to be nonpathogenic for a long time before the infections caused by this microorganism were confirmed [1]. Its threat to health remained unclear until the outbreak of nosocomial *S. marcescens* infections in the late 20th century [2,3]. Even though this organism exhibits a wide range of virulence factors and is relatively weak in virulence, it can infect critically ill or immunocompromised patients, as well as infants and newborns [4,5,6]. Researchers have pointed out that this microorganism could cause multiple infections, including meningitis, pneumonia, septicemia, and urinary tract infections, associated with poor clinical outcomes [7,8,9,10]. Due to their capability of adherence to invasive hospital equipment and forming biofilm, nosocomial infections caused by *S. marcescens* were difficult to treat [11,12]. What is more, these strains were usually resistant to ampicillin, ampicillin-sulbactam, amoxicillin, amoxicillin-clavulanate, narrow-spectrum cephalosporins, cephamycins, cefuroxime, nitrofurantoin, and colistin according to the Clinical and Laboratory Standards Institute (CLSI) guidelines. The increasing multidrug-resistant *S. marcescens* in the nosocomial environment has become a major concern. The situation worsens when this species acquires the ability to resist the last-resort antibiotics, such as carbapenems, due to the transfer of resistance genes, for example, *bla*_KPC_ was reported [13,14,15]. Now, the World Health Organization (WHO) has designated it as a research priority for developing alternative antibacterial strategies.

Beta-Lactam antibiotics have been popular as therapeutic drugs for over 70 years, which has led to an abundance of β-lactam-inactivating β-lactamases. Beta-lactamases, including plasmid-mediated extended-spectrum β-lactamases, AmpC cephalosporinases, and carbapenemases, are now present globally with some variants preferred in particular regions. A significant challenge in modern medicine has been the presence and dissemination of carbapenemases in *Enterobacteriaceae* since this hydrolase can decompose penicillins, cephalosporins, monobactams, and carbapenems [16,17]. IMP-26 was first identified and characterized as one kind of metallo-beta-lactamases (MBLs) from a *Pseudomonas aeruginosa* isolated in Singapore in 2010 [18]. The protein resembles IMP-4 but differs by one amino acid (Phe49Val). It displayed higher carbapenem hydrolysis activity toward meropenem than IMP-1 [19]. Since then, such MBLs have been reported in a variety of microbes worldwide, including *Enterobacteria* [20,21], *P. aeruginosa* [19,22], and *Klebsiella pneumoniae* [23]. As reported previously, the *bla*_IMP-26_ gene was found on various microbial chromosomes; only four articles ever described its location on a plasmid [24,25,26,27].

Colistin is one of the last therapeutic options for infections caused by multidrug-resistant Gram-negative bacteria [28]. However, a plasmid-mediated colistin resistance gene, *mcr-1*, was first identified in Chinese *Escherichia coli* isolates in 2016 [29]. In the years following the first description, several reports have described the emergence of *mcr-1* to *mcr-10* in different host species and geographic locations [30,31,32]. The *mcr-9* gene was found in a colistin-exposed *Salmonella* Typhimurium in 2019 [33]. It shared 65% and 63% amino acid identities with the closest relatives, MCR-3 and MCR-7, and between 33% and 45% with the other MCRs. Since then, the *mcr-9* gene has been identified in 40 countries across six continents [34]. These genes encode phosphoethanolamine transferase enzymes responsible for adding phosphoethanolamine to lipid A, which leads to a diminished affinity for colistin and antibiotic resistance [31].

Mobile elements are common in prokaryotic genomes and crucial for the evolution of plasmids and bacteria [35]. Typically, the evolution of a plasmid driven by transposons or IS elements usually results in structural changes through homologous recombination [36]. Porse et al. discovered that IS*26* mediated a large-scale deletion of a plasmid’s conjugation machinery, leading to a reduction in the plasmid fitness cost in *E. coli* hosts, improving the plasmid–host adaptation [37]. IS*26* disseminated antibiotic resistance genes in two distinct ways which differ from other non-IS*6* family members, disseminating antibiotic resistance genes in two specific ways. The first way is achieved through an IS*26*-flanked structure creating a cointegrate formation with duplication of the IS*26* and generation of a target duplication. The second way is forming a non-replicating circular intermediate containing a single IS*26* named a translocatable unit (TU). The IS*26* in TU targets an existing copy of IS*26* on the receptor’s sequence and adjacent to it without increasing the number of IS*26* copies or making a further duplication of the target [38,39]. Another noteworthy mobile element was Tn*3* [40]. Tn*3* transposons are a big and widespread transposon family allowing assembly, diversification, and redistribution of antimicrobial resistance genes, contributing to the transport of bacterial resistance genes among bacteria. They transpose in a “copy-and-paste” way in which the donor and target molecules are fused by repeated transposon copies [41].

Here, we characterized an IncHI2/2A plasmid harboring *bla*_IMP-26_ and *mcr-9* in a multidrug-resistant *S. marcescens* which displayed resistance to carbapenems and further studied the evolutionary pathway of such epidemic megaplasmids carrying *bla*_IMP-26_ mediated by IS*26* and Tn*3*.

## 2. Results

### 2.1. Antimicrobial Susceptibility Profiles

MICs obtained by the broth micro-dilution method are shown in Table 1. The isolate was susceptible only to amikacin according to CLSI breakpoints of 2021 (Table 1).

### 2.2. Genome Sequencing of S. marcescens YL4

*S. marcescens* YL4 possessed a circular chromosome with 5,171,477 bp and two plasmids (pYL4.1 and pYL4.2) with 316,459 bp and 46,771 bp, respectively. The GC content of chromosomes and plasmids was 59.30%, 47.64%, and 53.11%. On the YL4’s chromosome, 88 tRNA, 22 rRNA, 38 sRNA, and 4762 open reading frames were annotated using the Prokaryotic Genomes Annotation Pipeline server. In addition, 2 prophages, 1 CRISPR array, and 27 insertion elements were detected on the chromosome. ResFinder server analysis showed that the YL4 strain chromosome contains three resistance genes, including one beta-lactam resistance gene (*bla*_SRT-1_), aminoglycoside resistance gene (*aac(6′)-Ic*), and Tetracycline resistance gene (*tet(41)*) (Table 2).

### 2.3. Characteristics of the IncHI2/2A Plasmid pYL4.1

**Figure 1:**
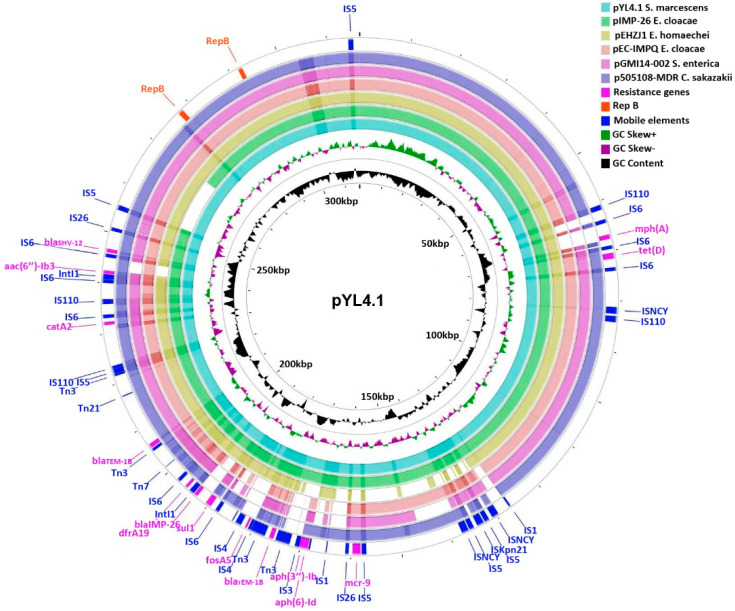
Genetic features of the pYL4.1 plasmid. Inner and outer circles correspond to average G+C content (black circle) and GC skew information (green and purple circles, respectively). The colored circles represent different plasmids (details are in the legend), and the Genbank numbers are as follows: pIMP26 (MH399264), pEHZJ1 (CP033103), pEC-IMPQ (EU855788), pGMI14-002 (CP028197), p505108-MDR (KY978628). An outer cyan-blue circle shows where resistance genes and *IntI* are located. The location of the resistance genes (magenta), insert elements (blue), and replication origins (orange) are also shown outside the round and tagged by different colors.

The IncHI2/2A plasmid pYL4.1’s DNA sequence comprises 316,459 bp with a G+C content of 47.64%. There are two replicons on the plasmid. One was 876 bp (291,621..292,496) in size; the other was 1056 bp (227,123..278,178) in length. BLAST search showed the backbone regions of pYL4 similar to pIMP-26 (Genbank ID: MH399264) [25], pEHZJ1 (Genbank ID: CP033103) [26], pEC-IMPQ (Genbank ID: EU855788) [42], pGMI14-002 (Genbank ID: CP028197), and p505108-MDR (Genbank ID: KY978628) (Figure 1). 

### 2.4. Gene Environments of bla_IMP-26_

**Figure 2:**
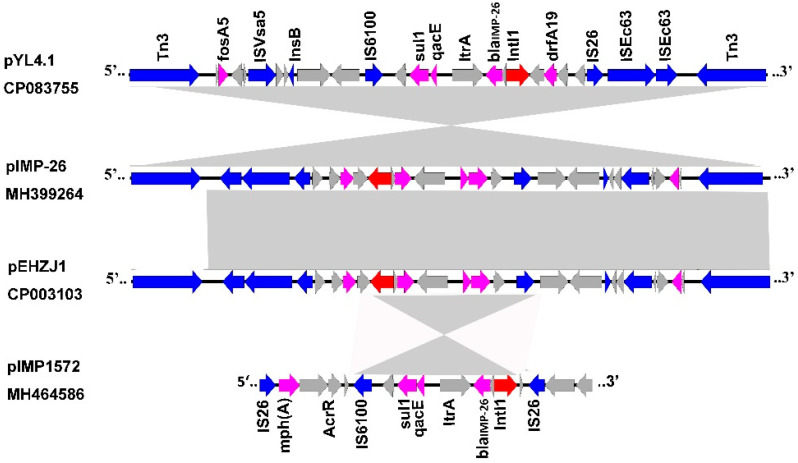
Genetic content of *bla*_IMP-26_. Comparison of the genetic environment of *bla*_IMP-26_ between different plasmids. Magenta arrows indicate resistance genes, and blue arrows indicate insertion sequences. Shading regions denote homologous regions (>95% nucleotide identity).

The genetic environment of *bla*_IMP-26_ reveals that it was partitioned into the class 1 integron cassette, sequentially arranged as *sul1-qacEΔ1-ItrA-bla*_IMP-26_*-Int1*. It contained a 5′-conserved segment (5′-CS) adjacent to IS*6100*, *InsB*, *ISVsa5*, *fosA5*, Tn*3*, *bla*_TEM-1B_, Tn*3*, and the 3′-CS was abutted to *drfA19*, mobile elements (IS*26*, couple of IS*Ec63*, *Tn2,* the last two belong to Tn*3* family), *bla*_TEM-1B_. The surrounding of *bla*_IMP-26_ in pYL4.1 was similar to but opposite to that of pEHZJ1 from *E. hormaechei* ST1103 in Zhejiang (accession: CP033103) and pIMP26 from *E. cloacae* RJ702 in Shanghai (accession: MH399264). The three highly similar plasmids share most genetic structures outside the class 1 integron cassette, for example, insertion elements (IS*26,* IS*Ec63,* IS*4,* IS*1,* Tn*3*) and resistance genes (*fosA5, bla*_TEM-1B_). Two Tn*2* transposons flank the multiple resistant regions, one on either end, and could be combined to create a composite transposon capable of moving as a single unit. Additionally, the Tn*3* family transposon at the 5′-CS of pEHZJ1 was IS*Ec63*, a transposon of the Tn*3* family, but not Tn*2*. Moreover, we found a *bla*_IMP-26_ containing plasmid pIMP1572 from *K. pneumoniae* KP-1572 (accession: MH464586) which differed from pYL4.1. The two plasmids only share the class 1 integron cassette region, and they do not share the other resistance genes and other elements. Importantly, although there is more or less a difference between the four plasmids, the *bla*_IMP-26_ is always located in the class 1 integron cassette, *IntI1-bla*_IMP-26_*-ORF1-qacE△1-sul1*, which consists of a complete 5′-conserved sequence (5′-CS, integrase *intl1*) and 3′-CS (*qacE△1-sul1*). It infers that class 1 integron may be significant for the transmission of *bla*_IMP-26_ between plasmids (Figure 2).

### 2.5. Gene Environments of Mcr-9

**Figure 3:**
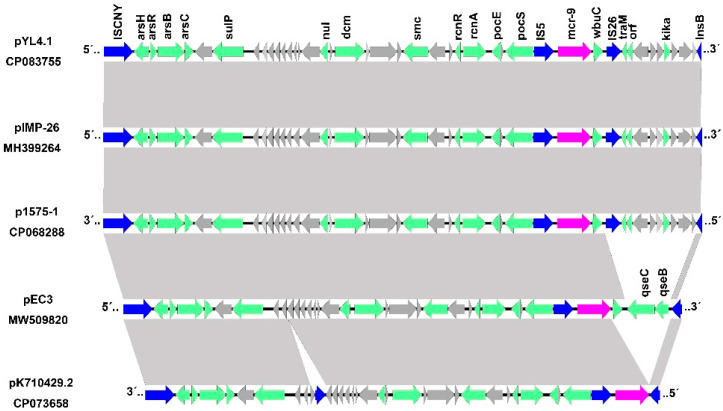
Comparison of the genetic environment of *mcr-9* in different plasmids. Magenta arrows indicate resistance genes, blue arrows indicate insertion sequences, green arrows indicate identified ORFs, and gray arrows represent hypothetical genes. Shaded regions denote homologous regions (>95% nucleotide identity).

We compared three *mcr-9* harboring plasmids reported in the last two years with our target plasmids. The four plasmids were pIMP-26 from *Enterobacter cloacae*, p1575-1 from *E. hormaechei* (accession: CP068288) [43], pEC3 from *E. coli* (accession: MW509820) [44], and pK714029-2 from *K. pneumoniae* (accession: CP073658) [45]. Comparing *mcr-9*-possessing regions among pYL4.1, pIMP-26, and p1575-1 revealed that they shared the same areas flanked by two mobile elements (from IS*CNY* to *InsB*) with 100% coverage and 98.88% identity. In all five plasmids, the upstream sequences of *mcr-9* were highly homologous, except for pK710429.2, which contained an insertion of the *InsB*. The structure of *rcnR-rcnA-pcoE-pcoS-*IS*903-mcr-9-wbuC* is present on all four plasmids, providing further evidence that these core elements perform a significant role in the conjugation and recombination processes of *mcr-9*. There are some unique sequences evident in the downstream line. Regulatory genes *qseC* and *qseB*, crucial to colistin resistance induction, were typically absent among the four observed plasmids, excluding pEC (Figure 3). However, the *mcr-9* gene expression did not increase after pretreatment with colistin in *qseC-qseB* carrying pEC [44]. In contrast, up-regulation of the *mcr-9* gene following colistin treatment was observed in pK714029-2 [45], the plasmid lacking *qseC-qseB*. The evidence suggests that the effect of *qseC-qseB* on *mcr-9* induction might differ among isolates with diverse genetic backgrounds [46]. Some additional genes may play a prominent role in *mcr-9* stimulation [34,47]. Further studies will be necessary to verify whether the *qseC-qseB* module or the other genes are essential for *mcr-9* induction.

### 2.6. Evolutionary Pathway of Megaplasmids IMP-26

**Figure 4:**
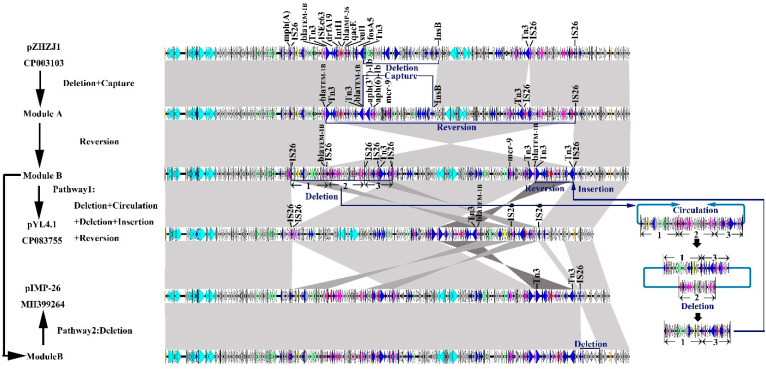
Evolutionary path between pEHZJ1 (CP033103), pYL4 (CP083755), and pIMP-26 (MH399264). The arrows indicate ORFs that have been confirmed or putatively confirmed and their orientations. Length of the arrow corresponds to the predicted ORF. The color code represents: replication initiation protein genes (orange), integrase genes (red), transposase genes and insertion elements (blue), antibiotic resistance genes (magenta), toxin-antitoxin system genes (yellow), heavy metal resistance genes (green), conjugal transfer genes (cray), putative, hypothetical, and other genes are represented by gray arrows. Shaded gray areas denote homologous regions with up to 95% nucleotide identity. When the shadow areas overlap, the color of the shaded region is darkened.

We compared pYL4.1’s whole sequence to the two most similar plasmids, pIMP-26 with 95% query coverage and 99.99% nucleotide identity and pEHZJ1 with 79% query coverage and 99.99% nucleotide identity. After a comprehensive comparison with all megaplasmids with *bla*_IMP-26_, the evolutionary path was hypothesized as below (Figure 4). For the pEHZJ1-derived module, a deletion of a 54 kb region followed by the acquisition of the *mcr-9* harboring multi-resistant region (MRR) and another acquisition of the *IntI1* area that occurred in pEHZJ1 generated a hypothetical module A plasmid. In the second step, a 179 kb MRR flanked by IS*26* and Tn*3* on module A reversed and developed a critical theoretical module B, from which pYL4 and pIMP-26 evolved in different pathways. A series of restructuring events occurred in the evolution from module B to pYL4.1. First, an IS*26* flanking MRR shear from module B formed a circle by connecting IS*26* on each end of the fragment. Second, deletion of a class 1 integron also flanked by IS*26* was conducted, creating a new component. Next, this fragment was reinserted between Tn*3* and IS*26*, located downstream of module B. The recombination was followed by a reversion of the *bla*_IMP-26_ harboring region backed by Tn*3*. In contrast, another evolutionary pathway from module B to pIMP-26 was simple, with a 13 kb hypothetical protein deletion behind IS*5* on the tail of module B and evolving into pIMP-26.

### 2.7. A Phage-Like Plasmid pYL4.2 Carrying bla_TEM-1B_

**Figure 5:**
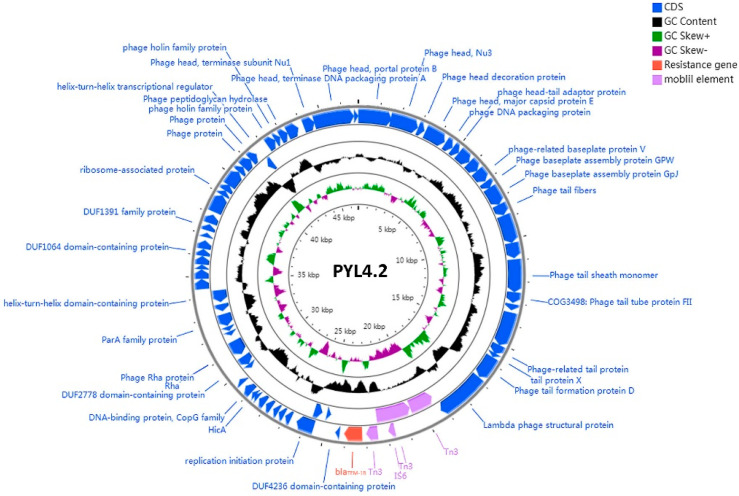
Structure of phage-like plasmid pYL4.2 carrying *bla*_TEM-1B_ from *S. marcescens* isolate YL4. The arrows with different colors represent open reading frames (ORFs), with purple, orange, and blue representing mobile elements, resistance genes, and ORFs, respectively. Inner and outer circles correspond to average G+C content (black circle) and GC skew information (green and purple circles), respectively.

Interestingly, the *S. marcescens* YL4 harbors another small plasmid, pYL4.2, a phage-like plasmid containing a large proportion of phage-related sequences, 46,771 bp in the plasmid sequence, with a 53.10% GC content. A fragment of ~8 kb in the plasmid was identified as the genome sequence of *S. marcescens* strain CBS12 isolated from contaminated platelet concentrates obtained from a Canadian donor. No more fragments are found similar to pYL4.2. Plasmid pYL4.2 belongs to the IncFII group and contains 74 ORFs. Noteworthy, the pYL4.2 genome encodes 47 proteins related to phages, such as coat protein, tail fiber protein, portal protein, terminator, outer membrane lytic protein, and others (Table 1). It suggests that pYL4.2 is a phage-like plasmid that carries many phage-like elements. A beta-lactamase encoding gene, *bla*_TEM-1B_, was identified on the pYL4.2, located downstream of a complete transposon Tn*3* and the insertion elements IS*26*. No additional antibiotic resistance genes were found in pYL4.2 (Figure 5).

## 3. Discussion

*S. marcescens* is an opportunistic nosocomial pathogen and is intrinsically multidrug resistant, mainly due to the presence of a large number of efflux pump genes [48,49], with the ability to produce myriad extracellular enzymes as well as metabolites which make it capable of adapting to both hostile and changing environments [50,51,52]. In *S. marcescens*, the PhoPQ system can regulate the expression of the *arn* operon by sensing polymyxin B (PB) and Mg^2+^, which results in the LPS being modified and leads to PB resistance [53,54]. Another important feature of *S. marcescens* is that pathogens can acquire antibiotic resistance genes from the surroundings rapidly, mainly due to the acquisition of plasmids and resistance genes [52], for example, *bla*_KPC_, as reported [13,14,15]. 

Here, we found the *S. marcescens* isolate YL4 possessed three drug-resistance genes on the chromosome, including beta-lactam resistance gene (*bla*_SRT-1_), aminoglycoside resistance gene (*aac(6′)-Ic*), and tetracycline resistance gene (*tet(41)*), together with two plasmids harboring a resistance gene. In addition, we found a plasmid-mediated colistin resistance gene, *mcr-9*, from a plasmid captured by the YL4 strain. This gene encodes a phosphoethanolamine transferase, contributing to narrowing the negative charge of the outer membrane of bacteria, and attenuates the affinity for colistin via the phosphoethanolamine incorporation into lipid A’s phosphate group, which results in colistin resistance [31]. It was suspected that *mcr-9* gene entered the *S. marcescens* YL4 by plasmid transfer, which is unnecessary for the survival of *S. marcescens*, bacteria intrinsically resistant to colistin [52]. The phenomenon portends a worrying prospect that the *mcr-9* gene may induce colistin resistance in other non-colistin resistant bacteria by horizontal gene transfer as reported [45].

pYL4.1 is a megaplasmid, which belongs to IncHI2/2A plasmid. The entire gene analysis revealed that the plasmid pYL4.1 was a close match to two *bla*_IMP-26_ harboring plasmids, pIMP-26 from *E. cloacae* RJ702 in Shanghai (accession: MH399264) [25] and pEHZJ1 from *E. hormaeechei* in Zhejiang (accession: CP033103) [26] reported before. An evolutionary path from pEHZJ1 to pYL4.1 or from pEHZJ1 to pIMP-26 was hypothesized. We assumed an IS*26*-flanked MRR experienced a process with shear, circulation, deletion, and insertion from module B to pYL4.1. This hypothesis is built on the theory posted by Harmer CJ et al. IS*26* transmits drug-resistance genes in two distinct forms, a couple of IS*26*s flanked cointegrate formation or single IS*26* containing a translocatable unit (TU) [38]. Past reports have confirmed that IS*26* is associated with the transfer of antibiotic resistance genes for its capability of forming translatable units. The translatable unit can be excised from the chromosome and reinserted into it. As a result, a tandem array is created and the number of copies of the resistant gene increases, which does not involve any fitness cost but does increase resistance to drugs [55,56]. Other deletions and reversion are closely related to IS*26* and another mobile element, Tn*3*, a transposon using a “copy-and-paste” mechanism to transfer gene fragments [41]. It is speculated that IS*26* and Tn*3* participation in the plasmid reorganization from clinical strains are likely to promote the spread of resistance genes among *S. marcescens* and other *Enterobacterales.*

Furthermore, the genetic content of *bla*_IMP-26_ was analyzed, and the results show that the *bla*_IMP-26_ was closely followed by *IntI1*. The *bla*_IMP-26_ harboring a class 1 integron cassette, sequentially arranged as *sul1-qacEΔ1-ItrA-bla*_IMP-26_*-Int1*, were identical to the other three plasmids reported before (pEHZJ1 from *E. hormaechei* ST1103 (accession: CP033103) [26], pIMP-26 from *E. cloacae RJ702* (accession: MH399264) [25], pIMP1572 from *K. pneumoniae* KP-1572 (accession: MH464586)) [27]. It suggests that the transmission of *bla*_IMP-26_ between plasmids may be mediated by and dependent on a class 1 integron, a mobile genetic component responsible for the transmission of multiple drug resistance [57]. These elements are capable of capturing, mobilizing, and integrating antibiotic-resistant gene cassettes [58]. Through lateral DNA transfer, they gained access to a variety of commensal and pathogenic bacteria and subsequently accumulate diverse antibiotic resistance genes [59,60]. The *bla*_IMP-26_ transfer mediated by class 1 integron might lead to the increase in carbapenem-resistant isolates, which are a risk to healthcare systems.

## 4. Materials and Methods

### 4.1. Bacterial Strain and Clinical Data

A 66-year-old female was hospitalized with pneumonia in Guangzhou, China, in February 2021. An injury to the central nervous system (CNS), type II respiratory failure, and type II diabetes were observed in the patient. *S. marcescens* was isolated from sputum samples collected during this patient’s hospitalization.

### 4.2. Bacterial Identification and Antimicrobial Susceptibility Testing

Antimicrobial susceptibility tests and biochemical identifications were performed using the Vitek 2 Compact system. The MIC values were determined using the broth micro-dilution methods. Results were interpreted according to guidelines set by the Clinical and Laboratory Standards Institute (CLSI) in 2021. The *S. marcescens* strain YL4 was used for further determinant analysis and antimicrobial element testing.

### 4.3. Whole-Genome Sequencing and Bioinformatics Analysis

According to the manufacturer’s instructions, genomic DNA was extracted using HiPure Bacterial DNA Kits (Magen, Guangzhou, China). Our libraries were constructed from a 350 bp small fragment genomic DNA library and a 10 kb fragment library. Genomic sequencing was performed using the Illumina Novaseq 6000 and Pacific Biosciences Sequel platforms (Guangzhou Gene Denovo Bioinformatics Technology Company, Guangzhou, China) to obtain short-read data and long-read data, respectively. Hybrid assembly was performed using Falcon (version 0.3.0) for long-read de novo assembly. The filtered short reads were utilized to correct the genome sequences to improve the quality of the assembly and determine the final genome sequences using Pilon (version 1.23). Genomic sequences were annotated using RAST 2.0 (http://rast.nmpdr.org/, accessed on 4 November 2021) and BLAST (https://blast.ncbi.nlm.nih.gov/Blast.cgi, accessed on 4 November 2021) to confirm the annotation results. PlasmidFinder was used to identify the plasmid (https://cge.food.dtu.dk/services/PlasmidFinder, accessed on 4 November 2021). ISFinder (https://www-is.biotoul.fr, accessed on 4 November 2021) and ResFinder (https://cge.cbs.dtu.dk/services/ResFinder, accessed on 4 November 2021) were used to identify resistance genes and insertion elements. Prophages were predicted by PHASTER (http://phaster.ca/, accessed on 15 November 2021). Plasmid sequence alignment to the GenBank database was performed using BLASTn (https://blast.ncbi.nlm.nih.gov/Blast.cgi, accessed on 4 November 2021).

### 4.4. GenBank Accession Numbers

The complete genome sequence of *S. marcescens* YL4 was submitted to GenBank under the accession number CP083754 (chromosome of *S. marcescens* YL4 strain), CP083755 (Plasmid pYL4.1), CP083756 (Phage like plasmid pYL4.2).

## 5. Conclusions

In summary, we first identified an IncHI2/2A plasmid carrying *bla*_IMP-26_ and *mcr-9* in a multidrug-resistant *Serratia marcescens* human isolate. After comprehensively comparing this plasmid and other relevant similar plasmids, we proposed an evolutionary pathway originating from ancestor pZHZJ1, including the acquisition of the *mcr-9* element and a few recombination events mediated by mobile factors, which resulted in the capture and loss of some drug-resistance genes. As widely known, mobile genetic elements play a crucial part in the transmission of antibiotic resistance genes between plasmids or between plasmids and strains’ chromosomes. Focusing on the evolutionary pathways between structurally similar plasmids is necessary in order to fully understand how multidrug-resistant plasmids evolve and move through microbial populations in diverse settings.

## Figures and Tables

**Table 1:** Antimicrobial susceptibility of *S. marcescens* YL4 isolate.

Antimicrobial Class	Antimicrobial Agents	MIC (μg/mL)	S	I	R
Cephalosporins	Cefepime	16	≤2	4–8	≥16
	Ceftazidime	≥64	≤4	8	≥16
	Cefatriaxone	≥64	≤1	2	≥4
β-lactam inhibitor combinations	Ticarcillin/clavulanate	≥128/2	≤16/2	32/2–64/2	≥128/2
Carbapenems	Imipenem	≥16	≤1	2	≥4
	Meropenem	≥16	≤1	2	≥4
	Ertapenem	≥8	≤0.5	1	≥2
Aminoglycosides	Tobramycin	≥16	≤4	8	≥16
	Amikacin	16	≤16	32	≥64
Fluorquinolones	Levofloxacin	4	≤0.5	1	≥2
	Ciprofloxacin	2	≤0.25	0.5	≥1
Sulfanilamides	Trimethoprim/sulfamethoxazole	≥16/304	≤2/38	-	≥4/76
Glycylcycline	Tigecycline	≥8	≤0.5	-	-
Monobactams	Aztreonam	≥64	≤4	8	≥16
Tetracyclines	Minocycline	≥16	≤4	8	≥16
	Doxycycline	≥16	≤4	8	≥16

**Table 2:** Antibiotic resistance genes in *S. marcescens* YL4.

Location	Antimicrobial Agents	Resistant Genes	Identity	Alignment Length/Gene Length	Start	End
Chromosome of YL4 strain (access No. CP083754)	Beta-lactam	*bla*_SRT-1_	96.22	1137/1137	775,741	776,877
	Tetracyline	*tet(41)*	93.57	1151/1182	1,054,107	1,055,257
	Aminoglycoside	*aac(6′)-Ic*	94.33	441/441	2,918,180	2,918,620
Plasmid pYL4.1 (access No. CP083755)	Aminoglycoside	*aph(6)-Id*	100.0	837/837	168,510	169,346
		*aph(3″)-Ib*	99.88	804/804	169,346	170,149
		*aac(6′)-Ib3*	100.0	555/555	241,940	242,494
	Polymyxin	*mcr-9*	100.0	1620/1620	158,061	159,680
	Fosfomycin	*fosA5*	100.0	420/420	181,153	181,572
	Macrolide	*mph(A)*	100.0	906/906	66,782	67,687
	Folate pathway antagonist	*sul1*	100.0	840/840	189,399	190,238
	Tetracycline	*dfrA19*	100.0	570/570	195,200	195,769
		*tet(D)*	100.0	1185/1185	70,462	71,646
	Beta-lactam	*bla*_TEM-1B_	100.0	861/861	175,551	176,411
		*bla*_IMP-26_	100.0	741/741	192,691	193,431
		*bla*_TEM-1B_	100.0	861/861	205,714	206,574
		*bla*_SHV-12_	100.0	861/861	245,958	246,818
	Quaternary ammonium compound	*qacE*	100.0	282/333	190,298	190,579
	Amphenicol	*catA2*	96.11	642/642	231,688	232,329
Phage-like plasmid pYL4.2 (access No. CP083756)	Beta-lactam	*bla*_TEM-1B_	100.0	861/861	23,202	24,062

## Data Availability

The complete genome sequence of *S. marcescens* YL4 was submitted to GenBank under the accession numbers CP083754 (chromosome of *S. marcescens* YL4 strain), CP083755 (Plasmid pYL4.1), CP083756 (Phage like plasmid pYL4.2).
